# Sex Steroids Modulate Uterine-Placental Vasculature: Implications for Obstetrics and Neonatal Outcomes

**DOI:** 10.3389/fphys.2016.00152

**Published:** 2016-04-26

**Authors:** Manuel Maliqueo, Bárbara Echiburú, Nicolás Crisosto

**Affiliations:** Endocrinology and Metabolism Laboratory, Department of Medicine West Division, School of Medicine, University of ChileSantiago, Chile

**Keywords:** placental angiogenesis, uterine blood flow, progesterone, androgen, estrogen

## Abstract

Adequate blood supply to the uterine-placental region is crucial to ensure the transport of oxygen and nutrients to the growing fetus. Multiple factors intervene to achieve appropriate uterine blood flow and the structuring of the placental vasculature during the early stages of pregnancy. Among these factors, oxygen concentrations, growth factors, cytokines, and steroid hormones are the most important. Sex steroids are present in extremely high concentrations in the maternal circulation and are important paracrine and autocrine regulators of a wide range of maternal and placental functions. In this regard, progesterone and estrogens act as modulators of uterine vessels and decrease the resistance of the spiral uterine arteries. On the other hand, androgens have the opposite effect, increasing the vascular resistance of the uterus. Moreover, progesterone and estrogens modulate the synthesis and release of angiogenic factors by placental cells, which regulates trophoblastic invasion and uterine artery remodeling. In this scenario, it is not surprising that women with pregnancy-related pathologies, such as early miscarriages, preterm delivery, preeclampsia, and fetal growth restriction, exhibit altered sex steroid concentrations.

## Introduction

During pregnancy, the placenta has important nutritional, metabolic, and endocrine functions that constitute the link between the mother and the fetus. The transfer of oxygen and essentials nutrients from maternal blood to the fetal bloodstream requires an adequate uterine perfusion and a placental vascular network. Abnormalities in these processes are associated with an increased risk for miscarriage, preterm delivery, preeclampsia, and fetal growth restriction (FGR) (Regnault et al., 2002).

The formation of blood vessels involves two consecutive processes: (1) vasculogenesis, which involves the structuring of primitive vessels from mesenchymal cells; and (2) angiogenesis, which is the generation of new blood vessels from preexisting vessels to form the vascular placental network (Charnock-Jones et al., 2004). Both processes are driven and regulated by multiple factors, including oxygen concentration, growth factors, cytokines, and steroid hormones. Sex steroids are essential to maintain a normal pregnancy, and they participate in the control of a wide range of maternal and placental functions as well as in the normal development of fetal organs such as the lungs and adrenal glands (Seaborn et al., 2010; Ishimoto and Jaffe, 2011). Moreover, variations in maternal serum concentrations of sex steroids have been described in conditions associated with abnormal placentation that impact placental perfusion, thus leading to pregnancy-related pathologies. Therefore, the aim of the present review is to summarize the current knowledge regarding the role of progesterone, androgens, and estrogens in the uterine-placental vasculature.

## Regulation of uterine vascular tone

During pregnancy, uterine blood flow increases dramatically mainly through a decrease in the uterine vascular resistance as a result of uterine arteries dilation and remodeling. Many of these effects are produced by changes in the muscular tone of uterine arteries that are mediated by the action of nitric oxide (NO) and prostanoids (prostacyclins, prostaglandins, and thromboxane). NO increases uterine blood flow through the relaxation of uterine arteries by a mechanism that involves a decrease in intracellular Ca^2+^ concentrations (i[Ca^2+^]) in vascular smooth muscle cells (VSMC). NO originates from the metabolism of L-arginine by the action of endothelial NO synthase (eNOS) in endothelial cells. Prostacyclin (PGI2) also induces vasodilation. However, has been observed that PGI2 exerts a compensatory action when NO production is reduced (Beverelli et al., 1997). Prostaglandin F2α (PGF2α) and thromboxane A2 (TXA2) induce vasoconstriction. Prostanoids are produced by the action of the cyclooxygenase (COX) enzymes, COX-1 and COX-2, on arachidonic acid. Of note, during pregnancy, serum concentrations of PGI2 increase dramatically, whereas PGF2α and TXA2 remain constant, thus favoring vasodilation (Mills et al., 1999).

Other regulators of the uterine vascular tone during pregnancy include adrenomedullin (Ross et al., 2010) and the components of the renin-angiotensin system, mainly angiotensin-(1–7) (Merrill et al., 2002). In rat uterine arteries, adrenomedullin induces relaxation mediated by the NO–cGMP-pathway (Ross et al., 2010). Angiotensin (1–7) is released from syncytiotrophoblasts, which act as a potent vasodilator in contrast to angiotensin II, which induces vasoconstriction (Valdes et al., 2006).

## Placental vasculature

**Figure 1:**
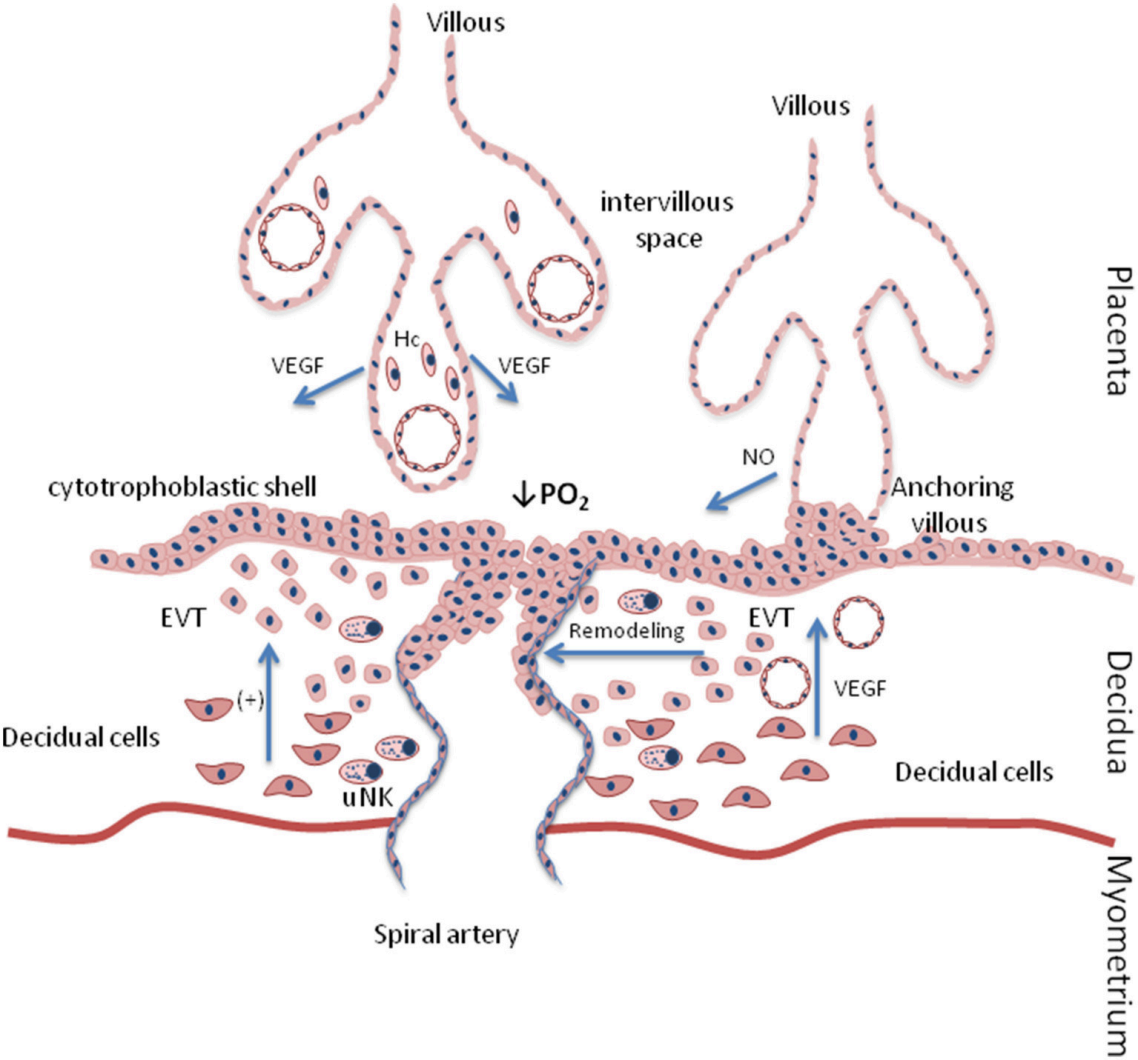
**Placental angiogenesis during early pregnancy**. The reaction of decidualization of stromal endometrial cells promotes the migration of extravillous cytotrophoblasts (EVT) across the decidua to reach the endothelial cells of the terminal segments of the uterine arteries occluding their lumen, which restricts the blood flow into the intervillous space and leads to reduced oxygen concentrations. Moreover, EVT remodel uterine spiral arterioles to increase maternal blood flow. On the other hand, trophoblastic cells, Hofbaur cells (Hc), and maternal decidual cells secrete VEGF, thus promoting angiogenesis. In addition, trophoblasts increase NOS activity, thus stimulating nitric oxide (NO) production and vasodilatation.

The placenta originates from the differentiation of trophoblastic cells from the pre-implantation embryo into cytotrophoblasts and syncytiotrophoblasts (Gerbaud and Pidoux, 2015). Two weeks after conception, the blastocyst cells acquire the ability to invade and migrate through the endometrial wall. The decidualization reaction of stromal endometrial cells subsequently results in an important increment in tissue permeability and vascular density. This reaction favors the migration of extravillous cytotrophoblasts (EVT) across the decidua to reach the endothelial cells of the terminal segments of the uterine arteries occluding their lumen (Figure 1), which restricts blood flow into the intervillous space and leads to a drop in oxygen concentration (Figure 1). Between weeks 11–12 until weeks 18–20 of gestation, EVT remodel the uterine spiral arteries. The remodeling allows the uterine spiral arteries to acquire a large capacitance and low resistance, thus gradually increasing maternal blood flow and oxygen levels (Rodesch et al., 1992).

The growth and development of the placental vascular network occurs through branching angiogenesis, which involves the formation of new vessels by the sprouting of preexisting vessels and a subsequent increase in the number of capillaries; it also occurs through non-branching angiogenesis, which involves the elongation of vessels and leads to the formation of capillary loops (Charnock-Jones et al., 2004).

The members of the vascular endothelial growth factor (VEGF) family are central in the regulation of placental vasculogenesis and angiogenesis (Demir et al., 2004). VEGF family members are produced by trophoblastic cells, Hofbaur cells, and maternal decidual cells (Figure 1) (Clark et al., 1996). The VEGF family has five members encoded by individual genes, including VEGF-A, VEGF-B, VEGF-C, VEGF-D, and PlGF (placenta growth factor). VEGF-A increases vascular permeability in endothelial cells, inducing placental vasculogenesis, and angiogenesis. Moreover, VEGF-A stimulates the expression of placental eNOS and NO production, thus inducing vasodilatation and promoting endothelial cell proliferation (Papapetropoulos et al., 1997) (Figure 1).

In general, VEGF-A, VEGF-B, and PlGF bind to VEGFR-1 (or Flt-1), whereas VEGF-A also binds to VEGFR-2 (or KDR). VEGF-A exhibits an increased affinity for Flt-1. However, KDR is more active in angiogenic stimulation (Stuttfeld and Ballmer-Hofer, 2009). In the human placenta, Flt-1 is located in syncytiotrophoblasts and endothelial cells of the placental villi (Helske et al., 2001). On the other hand, KDR is almost exclusively expressed in endothelial cells, which mostly occurs during the first trimester of gestation in parallel to the high angiogenic activity at that time (Yamazaki and Morita, 2006). The action of VEGF on angiogenesis is regulated by an impressive paracrine negative feedback system in which the soluble form of Flt-1 (sFlt-1) acts as a potent inhibitor of angiogenesis that is regulated by VEGF.

The hypoxic environment induces the expression of factors regulating the angiogenesis process, and hypoxia-inducible factor (HIF)-1α is one of the main factors (Kingdom and Kaufmann, 1999). Of note, villous trophoblasts cultured under hypoxic conditions (1% O_2_) express high levels of VEGF-A, Flt-1, and sFlt-1 mRNA (Munaut et al., 2008). Interestingly, recent evidence suggests that HIF-1α is also activated by non-hypoxic stimuli, such as growth factors, immunogenic cytokines, and sex steroids (Patel et al., 2010).

Other regulators of placental angiogenesis include angiopoietin (Ang)-1, Ang-2, and their receptor Tie-1. These proteins are complementary to the VEGF system but participate in the later stages of angiogenesis. In early pregnancy, Ang-2 is more highly expressed than Ang-1. However, Ang-2 decrease during the course of pregnancy (Geva et al., 2002). Finally, endoglin (Eng), a homodimeric transmembrane glycoprotein that belongs to the TFG-β (transforming growth factor beta) complex, contributes to placental angiogenesis; however, a placenta-derived soluble endoglin isoform (sEng) acts as an anti-angiogenic protein that inhibits TGF-β1 signaling in endothelial cells.

## Sex steroids and uterine vascular tone

The role of progesterone and estrogen in the regulation of the uterine vascular tone has been recognized for a long time. However, the effects of testosterone have only been recently addressed. In the placenta, androgens are metabolized to estrogens by the P450 aromatase. Dihydrotestosterone (DHT), which cannot be metabolized to estrogen, is subsequently reduced by aldo-keto reductase family 1 C into androstenediol (5α-androstane-3β, 17β-diol [3β-diol]), which has estrogen-like activity through ERβ (Lund et al., 2004). Therefore, androgenic and estrogenic effects cannot be easily separated in this tissue.

### Progesterone

Progesterone plays an important role in uterine vessel vasodilation before the 10th week of gestation (Dickey and Hower, 1996). This feature, along with the decreased resistance of the placental bed, contributes to a reduction of systemic blood pressure until 28 weeks of gestation.

Progesterone binds to its own receptors located in the nucleus and on the plasma membrane, mediating genomic, and non-genomic actions. In general, nuclear progesterone receptor (PR) comprises five isoforms, of which PR-A (81 kDa) and PR-B (116 kDa) are the most widely expressed in different tissues (Li and O'malley, 2003).

Progesterone has been implicated in the rapid increase of eNOS activity and the production of NO in human endothelial cells (Simoncini et al., 2007). In a similar manner, progesterone stimulates PGI2 production because it enhances the expression and activity of COX-1 and COX-2 (Hermenegildo et al., 2005). In addition, membrane progesterone receptors (mPRs) are present in VSMC, and they promote the decrease of the i[Ca^2+^] and lead to vasodilation (Minshall et al., 2002; Moussatche and Lyons, 2012). In this regard, in human umbilical vein endothelial cells (HUVECs), progesterone induces NO production through mPRα (Pang et al., 2015).

### Androgens

Testosterone promotes the proliferation of human myometrial microvascular endothelial cells through the activation of the MAPK/ERK-kinase pathway and VEGF-A production (Dietrich et al., 2011). In rats, the administration of androgen during pregnancy reduced uterine blood flow and elevated the maternal blood pressure due to an increased resistance of uterine vessels, which was due to the suppression of eNOS activity (Chinnathambi et al., 2013). Moreover, testosterone contracted the uterine arteries and reduced vascular relaxation due to the decline of endothelial NO production and the expression of prostacyclin and small conductance calcium-activated channel-3 (SK3). In contrast, hypoxia-responsive genes were increased, indicating poor uterine oxygenation induced by testosterone (Chinnathambi et al., 2014).

### Estrogens

Estrogens have an important function in the regulation of blood flow and microvascular volume because they control specific genes involved in vascular tone (Pastore et al., 2012). In this regard, NO is stimulated by both estrogen receptor isoforms (ERα and ERβ). Of interest, NO inhibition blunts the action of estradiol, suggesting that estrogen relaxation of myometrial arteries is mediated by both NO-dependent and -independent mechanisms (Rosenfeld et al., 1996). In addition, estrogens act on a 7-transmembrane G protein-coupled receptor named GPER. However, its activation reduces vascular tone in the rat uterus during pregnancy (Tropea et al., 2015).

Estradiol relaxes preconstricted human myometrial and placental arteries by binding to ERα and ERβ. However, this effect is lower than that in the myometrial than placental vessels (Corcoran et al., 2014). Moreover, estradiol can stimulate PGI2 synthesis due to upregulation of COX-1 expression, as observed in HUVEC and ovine fetal pulmonary artery cells. This effect is Ca^2+^ dependent and mediated by the activation of ERβ (Jun et al., 1998; Calkin et al., 2002; Sherman et al., 2002).

## Sex steroids and placental angiogenesis

The role of sex steroids in placental angiogenesis has not been widely studied. Preliminary evidence suggests that sex steroids can regulate both endometrial and placental angiogenesis.

### Progesterone

**Figure 2:**
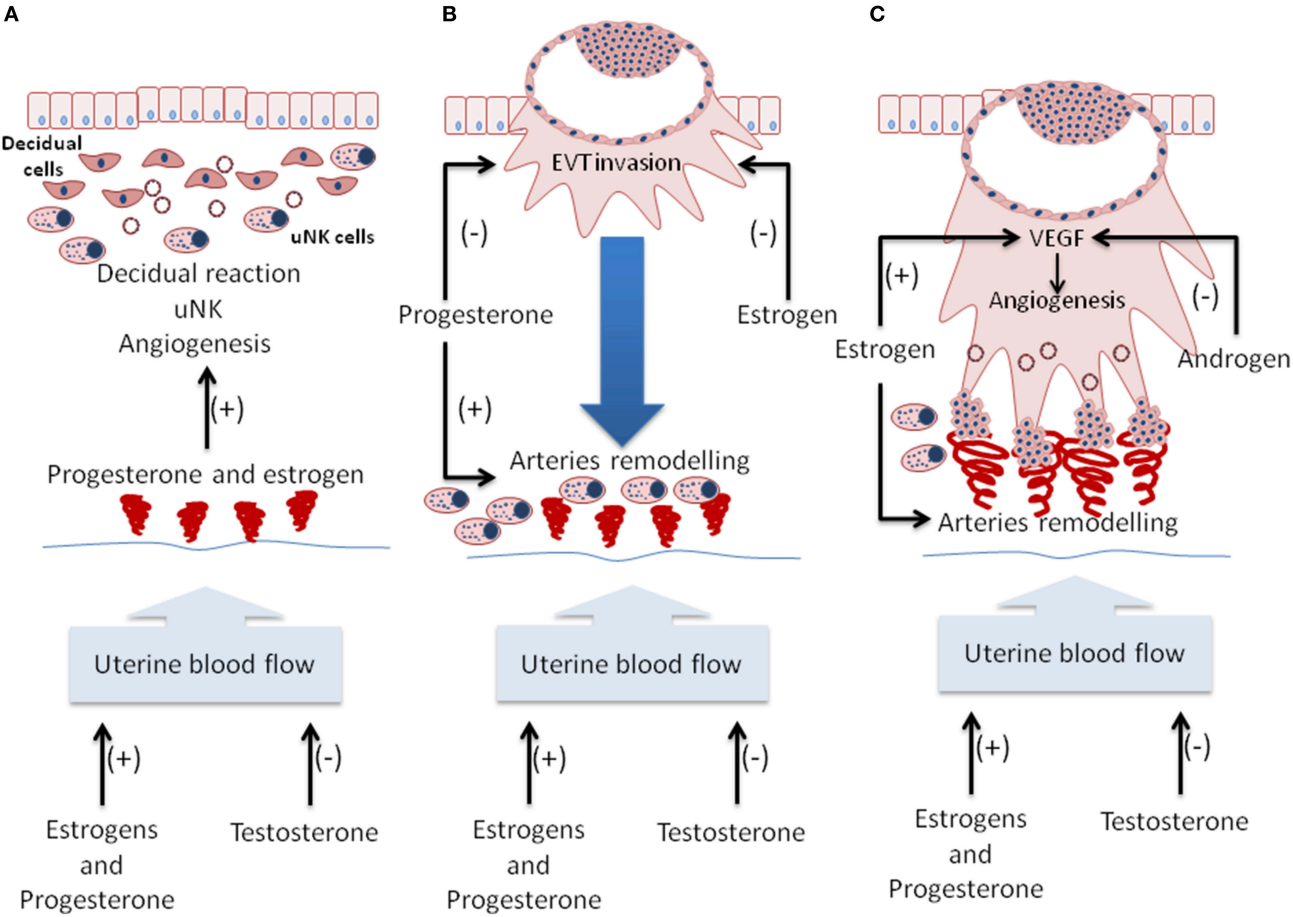
**Sex steroids regulate the uterine-placental vasculature**. **(A)** During secretory phase of the endometrial cycle, progesterone, and estrogen induce endometrial stromal decidualization to increase vascular permeability, recruit uterine natural killer cells (uNK), and increase endothelial cell proliferation. **(B)** During implantation, progesterone promotes remodeling of the arteries, most likely with the support of uNK. On the other hand, progesterone and estrogen regulate the invasiveness of extravillous trophoblast (EVT). **(C)** In early pregnancy, estrogen promotes the expression of vascular endothelial growth factor (VEGF), thus stimulating early placental angiogenesis. On the other hand, androgens inhibit the angiogenesis process. In addition, estrogen regulates the invasion of the uterine spiral artery by placental EVT. During the entire process, estrogen and progesterone increase uterine blood flow. However, testosterone reduces blood flow.

Progesterone has an important role in the activation of the decidual reaction in endometrial stromal cells, increasing the vascular permeability in the endometrial stroma through the activation of the nuclear receptor subfamily, group A, member 1 independently of VEGF action (Figure 2A; Goddard et al., 2014). Progesterone increases the number of uterine natural killer (uNK) cells (Bulmer and Lash, 2005), which most likely occurs indirectly through the decidualization reaction (Figure 2A). In this regard, uNK cells secrete a wide variety of angiogenic factors during early pregnancy, including VEGF-C, PlGF, Ang-1, Ang-2, and TGF-β1, promoting the vascular development of the decidua (Hanna et al., 2006).

In early pregnancy, PR is expressed in the endothelial cells of decidual tissue, and the binding of progesterone stimulates endothelial cell proliferation. This process is partly mediated by VEGF with no necessary estrogen priming (Wang et al., 1992). Moreover, progesterone regulates early trophoblast invasion because it reduces the invasive properties of EVT *in vitro* and the secretion of matrix metalloproteinase (MMP)- 2 and -9, which are primary mediators of vascular remodeling and angiogenesis in decidual tissue (Goldman and Shalev, 2006) (Figure 2B). However, progesterone promotes the migration of EVT by the upregulation of an insulin-like growth factor binding protein-1 and Dickkopf-related protein-1 (Halasz and Szekeres-Bartho, 2013). In addition, progesterone can promote the differentiation of a subfraction of decidual cells (named decidua-derived CD31^−^CD146^−^subfraction of side population (SP) cells) into endothelial cells and smooth muscle cells, suggesting that progesterone may play a role in the formation of new blood vessels in the placenta (Wang et al., 2013).

### Androgens

Androgen receptor is present in the cells of the syncytiotrophoblast and in the decidua during the first trimester of gestation (Horie et al., 1992). Rat models have shown that elevated androgen levels during pregnancy induce a reduction in placental weight and the activity of amino acid transporters (Sathishkumar et al., 2011; Sun et al., 2012). Moreover, androgens induce the downregulation of genes related to vascular development and angiogenesis (*Ccr3, Stra6, Dhcr7, Arid1a, Ptprj, Col1a2, Lef1, Col1a1*, and *Mmp2*) in the placenta (Figure 2C). Along with this antivasculogenic gene expression profile, the authors reported a reduction in radial and spiral artery diameters and branching angiogenesis (Gopalakrishnan et al., 2016). Thus, androgens could negatively regulate placental oxygenation, which is reflected by an increase in pimonidazole binding and HIF-1α levels (Gopalakrishnan et al., 2016).

### Estrogens

P450 aromatase is expressed in stromal uterine cells, indicating a local production of estrogen. Here, estrogen appears to facilitate decidualization and uterine neovascularization (Figure 2A), inducing the expression of HIF2α, Ang-2, Ang-4, and adrenomedullin (Das et al., 2009). Estrogen receptors (ER) α and β are expressed within villous trophoblasts of the human placentas (Bukovsky et al., 2003a,b). In this regard, the estrogen signaling has also been involved in the regulation of trophoblast differentiation and its invasive capacity in the hypoxic environment of the first trimester primate placenta. For example, similar to progesterone, estrogens act as regulators of the extent of remodeling during early pregnancy because they inhibit the invasive capacity of EVT (Figure 2B), reduce VEGF protein expression in the placenta anchoring villi and reduce the expression of integrins in cells from the anchoring villi and the cytotrophoblastic shell (Bonagura et al., 2012). Moreover, estradiol can regulate placental angiogenesis by the degranulation of mast cells that secrete important amounts of VEGF, suggesting a role of inflammation in this process. In this regard, estradiol and progesterone attract mast cells to the uterus (Corcoran et al., 2014).

In many species, including humans, estradiol induces the expression of the VEGF protein in the cytotrophoblast and increases the percent of vascularized area and vessel density in placental tissue (Albrecht et al., 2004; Robb et al., 2004; Albrecht and Pepe, 2010) (Figure 2C). In baboon cytotrophoblasts, VEGF mRNA increases in parallel with the increase in serum estradiol levels during early pregnancy (Hildebrandt et al., 2001). However, during the last two-thirds of pregnancy, the inhibition of P450 aromatase does not affect VEGF action in blood vessel development, suggesting that the cytotrophoblast loses its control by estrogen action during pregnancy (Albrecht and Pepe, 2010).

## Clinical implications

An abnormal blood supply to the uterine-placental region leads to early miscarriage, preterm delivery, preeclampsia, and FGR. In this regard, modifications to the circulating levels of sex steroids and/or uterine and placental sex steroids receptors are associated with poor obstetric and prenatal outcomes.

Women with unexplained recurrent pregnancy loss exhibit elevated uterine arterial impedance, which is negatively correlated with circulating progesterone levels. Of note, the administration of dydrogesterone, a synthetic progestin, reduced the resistance to blood flow in the uterine arteries, suggesting that insufficient progesterone action could be involved in a poor uterine blood supply and lead to miscarriage (Habara et al., 2002). Moreover, the elevated expression of Dickkopf-related protein-1 and low expression of PR-A have been observed in women with unexplained recurrent spontaneous miscarriage (Papamitsou et al., 2011; Bao et al., 2013). On the other hand, in growth-restricted pregnancies, PR expression in the placental tissue is positively correlated with IGF-1 expression and infant anthropometry, and it is independent of the presence of pregnancy pathologies (Akram et al., 2011). A group of studies has demonstrated that preeclampsia is associated with increased levels of progesterone along with increased expression of CYP11A, which inhibits trophoblastic proliferation and potentially the production of prostacyclin; this association affects the development of placental vasculature (Walsh and Coulter, 1989; He et al., 2013). Another group of studies demonstrated low circulating levels of progesterone and aldosterone in women with preeclampsia affected the secretion of endothelin-1, which is a potent vasoconstrictor. These results indicate that progesterone could be involved in the maintenance of normal blood pressure (Kiprono et al., 2013; Uddin et al., 2014). Therefore, normal development of placental vasculature is potentially dependent on physiological ranges of progesterone concentrations.

Because estrogen is an important regulator of uterine blood flow and the production of angiogenic factors in placental tissue, it is possible to hypothesize that estrogens are involved in the pathophysiology of pregnancy-related pathologies. At the 27th gestational week, estriol is positively associated with birth weight, birth length, and placental weight (Wuu et al., 2002). However, in rats, pharmacological doses of estradiol benzoate induce growth restriction, the reduction of placental weight, and trophoblastic degeneration (Matsuura et al., 2004). ERβ appears to be an important inducer of vasoconstrictor prostanoids because it increases the resistance of the feto-placental blood flow (Su et al., 2011).

Elevated androgen levels are a recurrent finding in preeclamptic women (Troisi et al., 2003; Salamalekis et al., 2006; Sharifzadeh et al., 2012), and this finding is likely related to a sex-related dysregulation in P450 aromatase (Steier et al., 2002; Sathishkumar et al., 2012). In women with polycystic ovary syndrome, which causes elevated androgen levels during pregnancy, the placenta presents an abnormal uterine blood flow as well as placentation with reduced endovascular trophoblast invasion (Palomba et al., 2010, 2012, 2013). Interestingly, placental tissues from these patients exhibit increased ERα expression (Maliqueo et al., 2015). Therefore, abnormalities observed in PCOS women could be attributed to estrogen or androgen action. However, it is not clear whether these alterations are directly associated with elevated androgen levels, as PCOS mothers also exhibit elevated insulin levels and a pro-inflammatory pattern (Sir-Petermann et al., 2007; Palomba et al., 2014), which may also influence placental function.

## Conclusions

Adequate uterine perfusion is necessary to achieve successful implantation. Moreover, placental vasculogenesis and angiogenesis ensure optimal transfer of oxygen and nutrients along with fetal detoxification. All these processes are essential for an adequate fetal development. The available data clearly note that sex steroids contribute to the modulation of uterine blood flow through the regulation of uterine vessels and placental vasculogenesis and angiogenesis, which involves controlling trophoblast invasion and the remodeling of uterine arteries.

## Author contributions

MM conceived and wrote the manuscript; BE and NC contributed with the writing and revised critically the manuscript.

## Funding

This work was supported by Fondo Nacional de Desarrollo Científico y Tecnológico (National Fund for Scientific and Technological Research; Fondecyt; Grant 11130250 and 11130126).

### Conflict of interest statement

The authors declare that the research was conducted in the absence of any commercial or financial relationships that could be construed as a potential conflict of interest.

## References

[B1] Akram S. K. Sahlin L. Ostlund E. Hagenas L. Fried G. Soder O. (2011). Placental IGF-I, estrogen receptor, and progesterone receptor expression, and maternal anthropometry in growth-restricted pregnancies in the Swedish population. Horm. Res. Paediatr. 75, 131–137. 10.1159/000320466 20962507

[B2] Albrecht E. D. Pepe G. J. (2010). Estrogen regulation of placental angiogenesis and fetal ovarian development during primate pregnancy. Int. J. Dev. Biol. 54, 397–408. 10.1387/ijdb.082758ea 19876841 PMC2804030

[B3] Albrecht E. D. Robb V. A. Pepe G. J. (2004). Regulation of placental vascular endothelial growth/permeability factor expression and angiogenesis by estrogen during early baboon pregnancy. J. Clin. Endocrinol. Metab. 89, 5803–5809. 10.1210/jc.2004-0479 15531545

[B4] Bao S. H. Shuai W. Tong J. Wang L. Chen P. Duan T. (2013). Increased Dickkopf-1 expression in patients with unexplained recurrent spontaneous miscarriage. Clin. Exp. Immunol. 172, 437–443. 10.1111/cei.12066 23600832 PMC3646443

[B5] Beverelli F. Bea M. L. Puybasset L. Giudicelli J. F. Berdeaux A. (1997). Chronic inhibition of NO synthase enhances the production of prostacyclin in coronary arteries through upregulation of the cyclooxygenase type 1 isoform. Fundam. Clin. Pharmacol. 11, 252–259. 10.1111/j.1472-8206.1997.tb00193.x 9243257

[B6] Bonagura T. W. Babischkin J. S. Aberdeen G. W. Pepe G. J. Albrecht E. D. (2012). Prematurely elevating estradiol in early baboon pregnancy suppresses uterine artery remodeling and expression of extravillous placental vascular endothelial growth factor and alpha1beta1 and alpha5beta1 integrins. Endocrinology 153, 2897–2906. 10.1210/en.2012-1141 22495671 PMC3359598

[B7] Bukovsky A. Caudle M. R. Cekanova M. Fernando R. I. Wimalasena J. Foster J. S.. (2003a). Placental expression of estrogen receptor beta and its hormone binding variant-comparison with estrogen receptor alpha and a role for estrogen receptors in asymmetric division and differentiation of estrogen-dependent cells. Reprod. Biol. Endocrinol. 1:36. 10.1186/1477-7827-1-36 12740031 PMC155643

[B8] Bukovsky A. Cekanova M. Caudle M. R. Wimalasena J. Foster J. S. Henley D. C.. (2003b). Expression and localization of estrogen receptor-alpha protein in normal and abnormal term placentae and stimulation of trophoblast differentiation by estradiol. Reprod. Biol. Endocrinol. 1:13. 10.1186/1477-7827-1-13 12646062 PMC151787

[B9] Bulmer J. N. Lash G. E. (2005). Human uterine natural killer cells: a reappraisal. Mol. Immunol. 42, 511–521. 10.1016/j.molimm.2004.07.035 15607807

[B10] Calkin A. C. Sudhir K. Honisett S. Williams M. R. Dawood T. Komesaroff P. A. (2002). Rapid potentiation of endothelium-dependent vasodilation by estradiol in postmenopausal women is mediated via cyclooxygenase 2. J. Clin. Endocrinol. Metab. 87, 5072–5075. 10.1210/jc.2002-020057 12414874

[B11] Charnock-Jones D. S. Kaufmann P. Mayhew T. M. (2004). Aspects of human fetoplacental vasculogenesis and angiogenesis. I. Molecular regulation. Placenta 25, 103–113. 10.1016/j.placenta.2003.10.004 14972443

[B12] Chinnathambi V. Balakrishnan M. Ramadoss J. Yallampalli C. Sathishkumar K. (2013). Testosterone alters maternal vascular adaptations role of the Endothelial NO system. Hypertension 61, 647–654. 10.1161/HYPERTENSIONAHA.111.00486 23339170 PMC3596870

[B13] Chinnathambi V. Blesson C. S. Vincent K. L. Saade G. R. Hankins G. D. Yallampalli C.. (2014). Elevated testosterone levels during rat pregnancy cause hypersensitivity to angiotensin II and attenuation of endothelium-dependent vasodilation in uterine arteries. Hypertension 64, 405–414. 10.1161/HYPERTENSIONAHA.114.03283 24842922 PMC4096063

[B14] Clark D. E. Smith S. K. Sharkey A. M. Charnockjones D. S. (1996). Localization of VEGF and expression of its receptors flt and KDR in human placenta throughout pregnancy. Hum. Reprod. 11, 1090–1098. 10.1093/oxfordjournals.humrep.a019303 8671397

[B15] Corcoran J. J. Nicholson C. Sweeney M. Charnock J. C. Robson S. C. Westwood M.. (2014). Human uterine and placental arteries exhibit tissue-specific acute responses to 17beta-estradiol and estrogen-receptor-specific agonists. Mol. Hum. Reprod. 20, 433–441. 10.1093/molehr/gat095 24356876 PMC4004081

[B16] Das A. Mantena S. R. Kannan A. Evans D. B. Bagchi M. K. Bagchi I. C. (2009). *De novo* synthesis of estrogen in pregnant uterus is critical for stromal decidualization and angiogenesis. Proc. Natl. Acad. Sci. U.S.A. 106, 12542–12547. 10.1073/pnas.0901647106 19620711 PMC2718343

[B17] Demir R. Kayisli U. A. Seval Y. Celik-Ozenci C. Korgun E. T. Demir-Weusten A. Y.. (2004). Sequential expression of VEGF and its receptors in human placental villi during very early pregnancy: differences between placental vasculogenesis and angiogenesis. Placenta 25, 560–572. 10.1016/j.placenta.2003.11.011 15135240

[B18] Dickey R. P. Hower J. F. (1996). Relationship of estradiol and progesterone levels to uterine blood flow during early pregnancy. Early Pregnancy 2, 113–120. 9363208

[B19] Dietrich W. Gaba A. Zhegu Z. Bieglmayer C. Mairhofer M. Mikula M.. (2011). Testosterone dependent androgen receptor stabilization and activation of cell proliferation in primary human myometrial microvascular endothelial cells. Fertil. Steril. 95, 1247–1255. e1241–1242. 10.1016/j.fertnstert.2010.11.012 21130428

[B20] Gerbaud P. Pidoux G. (2015). Review: An overview of molecular events occurring in human trophoblast fusion. Placenta 36(Suppl. 1), S35–S42. 10.1016/j.placenta.2014.12.015 25564303

[B21] Geva E. Ginzinger D. G. Zaloudek C. J. Moore D. H. Byrne A. Jaffe R. B. (2002). Human placental vascular development: vasculogenic and angiogenic (branching and nonbranching) transformation is regulated by vascular endothelial growth factor-A, angiopoietin-1, and angiopoietin-2. J. Clin. Endocrinol. Metab. 87, 4213–4224. 10.1210/jc.2002-020195 12213874

[B22] Goddard L. M. Murphy T. J. Org T. Enciso J. M. Hashimoto-Partyka M. K. Warren C. M.. (2014). Progesterone receptor in the vascular endothelium triggers physiological uterine permeability preimplantation. Cell 156, 549–562. 10.1016/j.cell.2013.12.025 24485460 PMC3985399

[B23] Goldman S. Shalev E. (2006). Difference in progesterone-receptor isoforms ratio between early and late first-trimester human trophoblast is associated with differential cell invasion and matrix metalloproteinase 2 expression. Biol. Reprod. 74, 13–22. 10.1095/biolreprod.105.044925 16135696

[B24] Gopalakrishnan K. Mishra J. S. Chinnathambi V. Vincent K. L. Patrikeev I. Motamedi M.. (2016). Elevated testosterone reduces uterine blood flow, spiral artery elongation, and placental oxygenation in pregnant rats. Hypertension 67, 630–639. 10.1161/HYPERTENSIONAHA.115.06946 26781277 PMC4752400

[B25] Habara T. Nakatsuka M. Konishi H. Asagiri K. Noguchi S. Kudo T. (2002). Elevated blood flow resistance in uterine arteries of women with unexplained recurrent pregnancy loss. Hum. Reprod. 17, 190–194. 10.1093/humrep/17.1.190 11756386

[B26] Halasz M. Szekeres-Bartho J. (2013). The role of progesterone in implantation and trophoblast invasion. J. Reprod. Immunol. 97, 43–50. 10.1016/j.jri.2012.10.011 23432871

[B27] Hanna J. Goldman-Wohl D. Hamani Y. Avraham I. Greenfield C. Natanson-Yaron S.. (2006). Decidual NK cells regulate key developmental processes at the human fetal-maternal interface. Nat. Med. 12, 1065–1074. 10.1038/nm1452 16892062

[B28] He G. Xu W. Chen Y. Liu X. Xi M. (2013). Abnormal apoptosis of trophoblastic cells is related to the up-regulation of CYP11A gene in placenta of preeclampsia patients. PLoS ONE 8:e59609. 10.1371/journal.pone.0059609 23555723 PMC3612086

[B29] Helske S. Vuorela P. Carpen O. Hornig C. Weich H. Halmesmaki E. (2001). Expression of vascular endothelial growth factor receptors 1, 2 and 3 in placentas from normal and complicated pregnancies. Mol. Hum. Reprod. 7, 205–210. 10.1093/molehr/7.2.205 11160848

[B30] Hermenegildo C. Oviedo P. J. Garcia-Martinez M. C. Garcia-Perez M. A. Tarin J. J. Cano A. (2005). Progestogens stimulate prostacyclin production by human endothelial cells. Hum. Reprod. 20, 1554–1561. 10.1093/humrep/deh803 15734756

[B31] Hildebrandt V. A. Babischkin J. S. Koos R. D. Pepe G. J. Albrecht E. D. (2001). Developmental regulation of vascular endothelial growth/permeability factor messenger ribonucleic acid levels in and vascularization of the villous placenta during baboon pregnancy. Endocrinology 142, 2050–2057. 10.1210/en.142.5.2050 11316772

[B32] Horie K. Takakura K. Imai K. Liao S. Mori T. (1992). Immunohistochemical localization of androgen receptor in the human endometrium, decidua, placenta and pathological conditions of the endometrium. Hum. Reprod. 7, 1461–1466. 1291578 10.1093/oxfordjournals.humrep.a137595

[B33] Ishimoto H. Jaffe R. B. (2011). Development and function of the human fetal adrenal cortex: a key component in the feto-placental unit. Endocr. Rev. 32, 317–355. 10.1210/er.2010-0001 21051591 PMC3365797

[B34] Jun S. S. Chen Z. Pace M. C. Shaul P. W. (1998). Estrogen upregulates cyclooxygenase-1 gene expression in ovine fetal pulmonary artery endothelium. J. Clin. Invest. 102, 176–183. 10.1172/JCI2034 9649571 PMC509079

[B35] Kingdom J. C. Kaufmann P. (1999). Oxygen and placental vascular development. Adv. Exp. Med. Biol. 474, 259–275. 10.1007/978-1-4615-4711-2_20 10635006

[B36] Kiprono L. V. Wallace K. Moseley J. Martin J. Lamarca B. (2013). Progesterone blunts vascular endothelial cell secretion of endothelin-1 in response to placental ischemia. Am. J. Obstet. Gynecol. 209, 44.e1–44.e6. 10.1016/j.ajog.2013.03.032 23545163 PMC4052216

[B37] Li X. O'malley B. W. (2003). Unfolding the action of progesterone receptors. J. Biol. Chem. 278, 39261–39264. 10.1074/jbc.R300024200 12893816

[B38] Lund T. D. Munson D. J. Haldy M. E. Handa R. J. (2004). Dihydrotestosterone may inhibit hypothalamo-pituitary-adrenal activity by acting through estrogen receptor in the male mouse. Neurosci. Lett. 365, 43–47. 10.1016/j.neulet.2004.04.035 15234470

[B39] Maliqueo M. Poromaa I. S. Vanky E. Fornes R. Benrick A. Akerud H.. (2015). Placental STAT3 signaling is activated in women with polycystic ovary syndrome. Hum. Reprod. 30, 692–700. 10.1093/humrep/deu351 25609240

[B40] Matsuura S. Itakura A. Ohno Y. Nakashima Y. Murata Y. Takeuchi M.. (2004). Effects of estradiol administration on feto-placental growth in rat. Early Hum. Dev. 77, 47–56. 10.1016/j.earlhumdev.2004.01.006 15113631

[B41] Merrill D. C. Karoly M. Chen K. Ferrario C. M. Brosnihan K. B. (2002). Angiotensin-(1-7) in normal and preeclamptic pregnancy. Endocrine 18, 239–245. 10.1385/ENDO:18:3:239 12450315

[B42] Mills J. L. Dersimonian R. Raymond E. Morrow J. D. Roberts L. J. II Clemens J. D.. (1999). Prostacyclin and thromboxane changes predating clinical onset of preeclampsia: a multicenter prospective study. JAMA 282, 356–362. 10.1001/jama.282.4.356 10432033

[B43] Minshall R. D. Pavcnik D. Browne D. L. Hermsmeyer K. (2002). Nongenomic vasodilator action of progesterone on primate coronary arteries. J. Appl. Physiol. (1985) 92, 701–708. 10.1152/japplphysiol.00689.2001 11796684

[B44] Moussatche P. Lyons T. J. (2012). Non-genomic progesterone signalling and its non-canonical receptor. Biochem. Soc. Trans. 40, 200–204. 10.1042/BST20110638 22260690

[B45] Munaut C. Lorquet S. Pequeux C. Blacher S. Berndt S. Frankenne F.. (2008). Hypoxia is responsible for soluble vascular endothelial growth factor receptor-1 (VEGFR-1) but not for soluble endoglin induction in villous trophoblast. Hum. Reprod. 23, 1407–1415. 10.1093/humrep/den114 18413304

[B46] Palomba S. Falbo A. Chiossi G. Orio F. Tolino A. Colao A.. (2014). Low-grade chronic inflammation in pregnant women with polycystic ovary syndrome: a prospective controlled clinical study. J. Clin. Endocrinol. Metab. 99, 2942–2951. 10.1210/jc.2014-1214 24873996

[B47] Palomba S. Falbo A. Russo T. Battista L. Tolino A. Orio F.. (2010). Uterine blood flow in pregnant patients with polycystic ovary syndrome: relationships with clinical outcomes. BJOG 117, 711–720. 10.1111/j.1471-0528.2010.02525.x 20236107

[B48] Palomba S. Russo T. Falbo A. Di Cello A. Amendola G. Mazza R.. (2012). Decidual endovascular trophoblast invasion in women with polycystic ovary syndrome: an experimental case-control study. J. Clin. Endocrinol. Metab. 97, 2441–2449. 10.1210/jc.2012-1100 22508703

[B49] Palomba S. Russo T. Falbo A. Di Cello A. Tolino A. Tucci L.. (2013). Macroscopic and microscopic findings of the placenta in women with polycystic ovary syndrome. Hum. Reprod. 28, 2838–2847. 10.1093/humrep/det250 23756703

[B50] Pang Y. Dong J. Thomas P. (2015). Progesterone increases nitric oxide synthesis in human vascular endothelial cells through activation of membrane progesterone receptor-alpha. Am. J. Physiol. Endocrinol. Metab. 308, E899–E911. 10.1152/ajpendo.00527.2014 25805192

[B51] Papamitsou T. Chatzistamatiou M. Grammatikopoulou D. Papadopoulou K. Lakis S. Economou Z.. (2011). Low expression of progesterone receptor A in intermediate trophoblast of miscarriages. Histol. Histopathol. 26, 609–614. 21432776 10.14670/HH-26.609

[B52] Papapetropoulos A. Garcia-Cardena G. Madri J. A. Sessa W. C. (1997). Nitric oxide production contributes to the angiogenic properties of vascular endothelial growth factor in human endothelial cells. J. Clin. Invest. 100, 3131–3139. 10.1172/JCI119868 9399960 PMC508526

[B53] Pastore M. B. Jobe S. O. Ramadoss J. Magness R. R. (2012). Estrogen receptor-alpha and estrogen receptor-beta in the uterine vascular endothelium during pregnancy: functional implications for regulating uterine blood flow. Semin. Reprod. Med. 30, 46–61. 10.1055/s-0031-1299597 22271294 PMC3674511

[B54] Patel J. Landers K. Mortimer R. H. Richard K. (2010). Regulation of hypoxia inducible factors (HIF) in hypoxia and normoxia during placental development. Placenta 31, 951–957. 10.1016/j.placenta.2010.08.008 20869770

[B55] Regnault T. R. Galan H. L. Parker T. A. Anthony R. V. (2002). Placental development in normal and compromised pregnancies-a review. Placenta 23 Suppl. A, S119–S129. 10.1053/plac.2002.0792 11978069

[B56] Robb V. A. Pepe G. J. Albrecht E. D. (2004). Acute temporal regulation of placental vascular endothelial growth/permeability factor expression in baboons by estrogen. Biol. Reprod. 71, 1694–1698. 10.1095/biolreprod.104.030882 15269101

[B57] Rodesch F. Simon P. Donner C. Jauniaux E. (1992). Oxygen measurements in endometrial and trophoblastic tissues during early-pregnancy. Obstet. Gynecol. 80, 283–285. 1635745

[B58] Rosenfeld C. R. Cox B. E. Roy T. Magness R. R. (1996). Nitric oxide contributes to estrogen-induced vasodilation of the ovine uterine circulation. J. Clin. Invest. 98, 2158–2166. 10.1172/JCI119022 8903336 PMC507661

[B59] Ross G. R. Yallampalli U. Gangula P. R. R. Reed L. Sathishkumar K. Gao H. J.. (2010). Adrenomedullin relaxes rat uterine artery: mechanisms and influence of pregnancy and estradiol. Endocrinology 151, 4485–4493. 10.1210/en.2010-0096 20631002 PMC2940500

[B60] Salamalekis E. Bakas P. Vitoratos N. Eleptheriadis M. Creatsas G. (2006). Androgen levels in the third trimester of pregnancy in patients with preeclampsia. Eur. J. Obstet. Gynecol. Reprod. Biol. 126, 16–19. 10.1016/j.ejogrb.2005.07.007 16139944

[B61] Sathishkumar K. Balakrishnan M. Chinnathambi V. Chauhan M. Hankins G. D. V. Yallampalli C. (2012). Fetal sex-related dysregulation in testosterone production and their receptor expression in the human placenta with preeclampsia. J. Perinatol. 32, 328–335. 10.1038/jp.2011.101 21904298 PMC3712643

[B62] Sathishkumar K. Elkins R. Chinnathambi V. Gao H. Hankins G. D. Yallampalli C. (2011). Prenatal testosterone-induced fetal growth restriction is associated with down-regulation of rat placental amino acid transport. Reprod. Biol. Endocrinol. 9:110. 10.1186/1477-7827-9-110 21812961 PMC3162507

[B63] Seaborn T. Simard M. Provost P. R. Piedboeuf B. Tremblay Y. (2010). Sex hormone metabolism in lung development and maturation. Trends Endocrinol. Metab. 21, 729–738. 10.1016/j.tem.2010.09.001 20971653

[B64] Sharifzadeh F. Kashanian M. Fatemi F. (2012). A comparison of serum androgens in pre-eclamptic and normotensive pregnant women during the third trimester of pregnancy. Gynecol. Endocrinol. 28, 834–836. 10.3109/09513590.2012.683061 22559844

[B65] Sherman T. S. Chambliss K. L. Gibson L. L. Pace M. C. Mendelsohn M. E. Pfister S. L.. (2002). Estrogen acutely activates prostacyclin synthesis in ovine fetal pulmonary artery endothelium. Am. J. Respir. Cell Mol. Biol. 26, 610–616. 10.1165/ajrcmb.26.5.4528 11970914

[B66] Simoncini T. Fu X. D. Caruso A. Garibaldi S. Baldacci C. Giretti M. S.. (2007). Drospirenone increases endothelial nitric oxide synthesis via a combined action on progesterone and mineralocorticoid receptors. Hum. Reprod. 22, 2325–2334. 10.1093/humrep/dem109 17545686

[B67] Sir-Petermann T. Echiburu B. Maliqueo M. M. Crisosto N. Sanchez F. Hitschfeld C.. (2007). Serum adiponectin and lipid concentrations in pregnant women with polycystic ovary syndrome. Hum. Reprod. 22, 1830–1836. 10.1093/humrep/dem090 17468256

[B68] Steier J. A. Ulstein M. Myking O. L. (2002). Human chorionic gonadotropin and testosterone in normal and preeclamptic pregnancies in relation to fetal sex. Obstet. Gynecol. 100, 552–556. 10.1097/00006250-200209000-00024 12220777

[B69] Stuttfeld E. Ballmer-Hofer K. (2009). Structure and function of VEGF receptors. IUBMB Life 61, 915–922. 10.1002/iub.234 19658168

[B70] Su E. J. Ernst L. Abdallah N. Chatterton R. Xin H. Monsivais D.. (2011). Estrogen receptor-beta and fetoplacental endothelial prostanoid biosynthesis: a link to clinically demonstrated fetal growth restriction. J. Clin. Endocrinol. Metab. 96, E1558–E1567. 10.1210/jc.2011-1084 21832119 PMC3200254

[B71] Sun M. Maliqueo M. Benrick A. Johansson J. Shao R. Hou L.. (2012). Maternal androgen excess reduces placental and fetal weights, increases placental steroidogenesis, and leads to long-term health effects in their female offspring. Am. J. Physiol. Endocrinol. Metab. 303, E1373–E1385. 10.1152/ajpendo.00421.2012 23047983

[B72] Troisi R. Potischman N. Roberts J. M. Ness R. Crombleholme W. Lykins D.. (2003). Maternal serum oestrogen and androgen concentrations in preeclamptic and uncomplicated pregnancies. Int. J. Epidemiol. 32, 455–460. 10.1093/ije/dyg094 12777436

[B73] Tropea T. De Francesco E. M. Rigiracciolo D. Maggiolini M. Wareing M. Osol G.. (2015). Pregnancy Augments G Protein Estrogen Receptor (GPER) induced vasodilation in rat uterine arteries via the Nitric Oxide - cGMP Signaling Pathway. PLoS ONE 10:e0141997. 10.1371/journal.pone.0141997 26536245 PMC4633123

[B74] Uddin M. N. Horvat D. Jones R. O. Beeram M. R. Zawieja D. C. Perger L.. (2014). Suppression of aldosterone and progesterone in preeclampsia. J. Matern. Fetal Neonatal Med. 28, 1296–1301. 10.3109/14767058.2014.951627 25164552

[B75] Valdes G. Neves L. A. A. Anton L. Corthorn J. Chacon C.. (2006). Distribution of angiotensin-(1-7) and ACE2 in human placentas of normal and pathological pregnancies. Placenta 27, 200–207. 10.1016/j.placenta.2005.02.015 16338465

[B76] Walsh S. W. Coulter S. (1989). Increased placental progesterone may cause decreased placental prostacyclin production in preeclampsia. Am. J. Obstet. Gynecol. 161, 1586–1592. 10.1016/0002-9378(89)90931-9 2513720

[B77] Wang J. D. Fu Y. Shi W. L. Zhu P. D. Cheng J. Qiao G. M.. (1992). Immunohistochemical localization of progesterone receptor in human decidua of early pregnancy. Hum. Reprod. 7, 123–127. 1551948 10.1093/oxfordjournals.humrep.a137545

[B78] Wang Q. Shen L. Huang W. Song Y. Xiao L. Xu W.. (2013). Vasculogenesis of decidua side population cells of first-trimester pregnancy. Stem Cell Res. Ther. 4:50. 10.1186/scrt200 23651491 PMC3706889

[B79] Wuu J. Hellerstein S. Lipworth L. Wide L. Xu B. Yu G. P.. (2002). Correlates of pregnancy oestrogen, progesterone and sex hormone-binding globulin in the USA and China. Eur. J. Cancer Prev. 11, 283–293. 10.1097/00008469-200206000-00012 12131662

[B80] Yamazaki Y. Morita T. (2006). Molecular and functional diversity of vascular endothelial growth factors. Mol. Divers. 10, 515–527. 10.1007/s11030-006-9027-3 16972015

